# Role of innate immune cells in multiple sclerosis

**DOI:** 10.3389/fimmu.2025.1540263

**Published:** 2025-02-17

**Authors:** Carolina Prado, Andrés A. Herrada, Daniel Hevia, Lorna Galleguillos Goiry, Noelia Escobedo

**Affiliations:** ^1^ Laboratorio de Neuroinmunología, Centro Científico y Tecnológico de Excelencia Ciencia & Vida, Fundación Ciencia & Vida, Santiago, Chile; ^2^ Facultad de Medicina y Ciencia, Universidad San Sebastián, Santiago, Chile; ^3^ Lymphatic Vasculature and Inflammation Research Laboratory, Instituto de Ciencias Biomédicas, Facultad de Ciencias de la Salud, Universidad Autónoma de Chile, Talca, Chile; ^4^ Center for Studies and Innovation in Dentistry, Facultad de Odontología, Universidad Finis Terrae, Santiago, Chile; ^5^ Neurology and Psychiatry Department, Clínica Alemana, Neurology and Neurosurgery Department, Clínica Dávila, Santiago, Chile

**Keywords:** innate immune cells, experimental autoimmune encephalomyelitis, multiple sclerosis, macrophages, dendric cells

## Abstract

Multiple sclerosis (MS) is a chronic autoimmune, inflammatory and neurodegenerative disease affecting the central nervous system (CNS). MS is associated with a complex interplay between neurodegenerative and inflammatory processes, mostly attributed to pathogenic T and B cells. However, a growing body of preclinical and clinical evidence indicates that innate immunity plays a crucial role in MS promotion and progression. Accordingly, preclinical and clinical studies targeting different innate immune cells to control MS are currently under study, highlighting the importance of innate immunity in this pathology. Here, we reviewed recent findings regarding the role played by innate immune cells in the pathogenesis of MS. Additionally, we discuss potential new treatments for MS based on targets against innate immune components.

## Introduction

Multiple sclerosis (MS) is an autoimmune disorder associated with significant neurodegeneration and neuroinflammation. This disease is triggered by an autoimmune response directed against myelin producing demyelinated areas in both the white and gray matter of the brain and spinal cord. These lesions indicate loss of myelin and myelin-producing oligodendrocytes, resulting in disrupted conduction of electrical impulses (1). MS can be divided into 3 subtypes: relapse remitting MS (RMSS), primary progressive MS, and secondary progressive MS (2). The most common type of MS is RRMS, which is characterized by recurring episodes of neurological dysfunction, followed by clinical recovery. The disease symptoms are heterogeneous and depend on the location of the lesions in the CNS and range from sensory disturbances, bladder dysfunction, cognitive deficits, limb weakness, ataxia, and fatigue (3).

Even though the pathogenesis of MS is not fully understood, there are many pieces of the puzzle that are starting to shape up, with factors that may induce a primary inflammatory disease or a primary oligodendroglial pathology followed by inflammation (4). The multifactorial and complex interaction between genetic and environmental factors plays an important role in triggering the disease. The prevalence of the MS-risk allele HLA-DR15 and many single nucleotide polymorphisms of genes that are important for the differentiation or effector function of pathogenic T cells strengthens the concept of immune-mediated disease with the contribution of different risk factors: childhood obesity (3, 5), cigarette smoking (6–8), Epstein-Barr virus infection (9–11), vitamin D deficiency (12–14) and night shift work at young age (15–18).

If we recapitulate in the actual biological knowledge, MS is thought to be caused by an autoimmune response towards central nervous system (CNS) self-antigens in genetically susceptible individuals, where autoreactive T cells are supposed to be the disease-initiating immune cells (19). In the last decades, B cells were recognized as crucial immune cells in this process, including antibody-dependent and independent effects in the compartmentalized inflammation (20–22). Also, myeloid cells are important contributors to the pathology, being central actors in the disease progression, cortical atrophy, neurodegeneration, and disability (23).

The acute inflammation consists of an invasion of monocytes and lymphocytes into white matter (WM), with a lesser degree into deep gray matter, with concomitant activation of microglia and astrocytes. This demyelination leads to the formation of acute focal WM lesions, formed by dense infiltration of myelin-laden macrophages, lymphocytes, and important axonal loss followed by neurological disorders and physical disabilities (24). Chronic inflammation results from diffuse glial activation at the rim of chronic active lesions, which can reach considerable distances into normal- appearing white matter, with predominant lymphocytic inflammation in the meninges and perivascular spaces. Meningeal inflammation is typically diffuse but may also form follicle-like structures (25).

The available therapies for MS aim to shift the immune cell repertoire from a pro-inflammatory towards an anti-inflammatory phenotype, involving regulatory T (Treg) and B cells (mainly) and anti-inflammatory macrophages (in clinical trials). Despite the clear association of dysfunctional T and B cells in MS, during the last years, mounting evidence of different innate immune cell types involved in the pathogenesis of MS has emerged. This review gives a conceptual overview on the different innate immune cell types involved in MS pathology, discussing potential new targets for treatment (26).

## Innate immune cells in MS

### Dendritic cells

Dendritic cells (DCs) are highly specialized antigen presenting cells (APCs) with the unique ability to stimulate naïve T cells. First discovered by Steinman et al. in 1973 and named because of their dendritic shapes (27), DCs are constantly sensing pathogen signals or damage-associated molecular patterns, patrolling in different anatomical locations (28). In this immature state, DCs have the ability to capture and process antigens in a very efficient way, while their capacity to activate naïve T cells is weak, because of the lower expression of costimulatory molecules and cytokines (29). Once DCs capture and process pathogens or damage-associated molecular patterns, DCs go through a maturation process, increasing the expression of costimulatory molecules and loading antigen fragments into the major histocompatibility complex (MHC), while they migrate into lymph nodes where they efficiently activate and differentiate effector T cells (29). Additionally, mature DCs in the presence of Interleukin (IL)-10 and IL-27 can block T cell activation and promote Treg expansion, inducing immune tolerance (30). According to the developmental origin, surface markers and transcriptome profiles, DCs can be divided into three major subsets: Conventional DCs (cDCs), which can be further divided into cDC1s and cDC2s, plasmacytoid DCs (pDCs) and monocyte-derived DCs (moDCs) (31, 32). During steady state, cDCs are distributed in lymph and non-lymphoid tissues, and they are the main APC among DCs subsets, with cDC1 activating CD8+ T cells and cDC2 mainly activating CD4+ T cells (33, 34). pDCs are mostly confined to lymphoid tissues in homeostatic conditions and are capable of rapidly responding to virus infection by producing high levels of type I interferons (IFNs) (33, 34). moDCs develop from monocyte DCs progenitor under inflammatory conditions, working together with cDCs in response to inflammation or infection (35). Because DCs act as a bridge between innate and adaptive immunity, they are key players in autoimmune processes such as MS.

Even though it was first believed that the CNS was an immunoprivileged site, early studies during 1990´s in rats showed the presence of DCs in the meninges and the choroid plexus in healthy conditions, and infiltration of DCs into the brain after inflammatory conditions, suggesting that DCs could be playing an important role in neuroinflammatory processes (36, 37). The use of transgenic animals expressing enhanced yellow fluorescent protein downstream of the DCs-associated CD11c promoter, confirmed the presence of a small population of DCs in the CNS in homeostasis and during different pathological conditions (38–40). An animal model commonly used to study MS immunopathology is the experimental autoimmune encephalomyelitis (EAE) mouse model. As with MS, EAE is accompanied by lesion formation and paralysis caused by immune cells invading the CNS. By using this model, Matyszaki et al. found infiltration of DCs, characterized by the expression of CD103 and MHC class II (MHCII), in different lesions, mostly in perivascular regions but some DCs were also found in the brain parenchyma (41). Soon after, DCs infiltration into perivascular regions and in lesser extent into parenchymal regions of brain and spinal cords were also observed in mice at the peak stage of EAE (42). Although it was clear that DCs are capable of invading the CNS during EAE, the contribution of DCs to induce neuroinflammation during EAE was unclear, with contradictory studies suggesting from one side that DCs inhibit T cell activation reducing neuroinflammation (43, 44), while other studies showing direct contribution of DCs to the induction and maintenance of neuroinflammation in EAE (45, 46), although different DCs maturation stages analyzed could in part explain these contradictory results. In fact, the intracerebral microinjection of DCs cultured in different medium conditions showed that fully mature DCs exacerbated the onset and clinical course of EAE, while intracerebral microinjection of semi-mature DCs delayed EAE symptoms (47). To try to clarify the role of DCs in EAE, the use of constitutively or inducible DCs-depleted mice models were developed, but again, contradictory results were obtained, with some groups showing that DCs depletion leads to the loss of tolerance to self-antigens and increased EAE symptoms while other studies showed no major effect of DCs depletion over EAE symptoms and progression (47–50). However, the finding that CD11c is expressed in other APCs such as microglia, monocytes and macrophages, makes it very difficult to distinguish the real contribution of DCs in EAE versus the other immune cell types in these CD11c-expressing cell ablation systems. To overcome this problem, single cell mapping technology and the use of new transgenic mice has been developed in the last years, to specifically identify different DCs subsets and to interrogate the contribution of each of these subsets in the development of EAE. Thus, by mass cytometry technique together with high-dimensional data mining, Mundt et al. found different APC populations, including cDC1, cDC2 and pDCs, specifically in the outer membrane of the meninges, the dura mater, at steady-state (51). Next, by using the Cx3cr1CreERT2 strain, that allows specific targeting of macrophages, monocytes or DCs depending on the time that tamoxifen is given, where early tamoxifen treatment target long-lived, self-maintaining cells such as microglia and macrophages, whereas late tamoxifen injection target also DCs and monocytes, this group found that cDCs, particularly cDC2 subset, but not microglia or macrophages, are necessary for the activation of T cells in the CNS and to promote EAE pathogenesis (51). By using a different approach based on single-cell RNA sequencing analysis (scRNAseq) of different CNS compartments, Jordão et al. analyzed multiple myeloid cell populations in steady-state as well as during different stages of EAE (52). Again, although DCs numbers are low at homeostatic CNS, this population increase during EAE, and reduction of MHCII levels in DCs decreased EAE severity, confirming a critical role of DCs during EAE (52). Mechanistically, C-X-C chemokine receptor type 1 (CXCR1) expression on DCs seems to be important in this process, since specific ablation of CXCR1 on DCs reduces EAE severity in part by reducing proinflammatory cytokine production by DCs (53). All these results highlight the role of DCs, specifically cDCs, as the main APC in the CNS during EAE and suggest that targeting DC function could be a good strategy to treat MS. In fact, a recent study showed that reducing cDC1 subset by the use of CXCR1-specific chimeric antigen receptor (CAR)-T cells, decreased EAE symptoms in a CD4+ T cell-induced passive EAE (54). Thus, targeting DCs subsets could be a good therapeutic strategy to treat MS, although more studies are needed to confirm these observations.

Soon after the observation of DCs in the CNS of different animal models of MS, two populations of DCs, myeloid and pDCs, were observed in the cerebrospinal fluid (CSF) from healthy volunteers with increased numbers of pDCs in the CSF of people with MS (pwMS) (55). In fact, pwMS during relapses showed an increased number of pDCs in the CSF (56). Increased number of DCs inside of spinal cord and brains from pwMS were observed, specifically in perivascular regions of MS lesions where they contained myelin components, potentially presenting antigens to CD8+ T cells (57). The increased number of DCs in MS lesions could be explained in part by the increased concentration of the chemokines monocyte chemotactic protein (MCP) -1, -2 and -3 and CXCL10 (58, 59). Additionally, increased expression of the chemokine regulated on activation normal T cell expressed and secreted (RANTES) and macrophage inflammatory protein (MIP)-1α/β in the CSF of pwMS have been detected, together with increased expression of their receptor CCR5 in peripheral cDCs of pwMS (60–62). Moreover, scRNAseq analysis of cells from CSF of RMSS patients showed the presence of cDCs and pDCs (63). A different single-cell analysis study showed an increased proportion of cDCs in the CSF compared to the blood from untreated relapsed pwMS (64). All these data suggest that the accumulation of different DCs subsets in perivascular regions of the CNS from pwMS could be contributing to the increase of neuroinflammation by actively presenting myelin antigens to T cells leading to their activation and the perpetuation of the damage and suggest that, targeting DCs could be a good strategy to reduce neuroinflammation in pwMS.

### Macrophages

Macrophages represent a heterogeneous group of immune cells that can phagocytose, playing a key role in the initiation, triggering, and resolution of an immune response, as well as repairing inflammation-damaged tissues (65). Macrophages are found in almost all the tissues in the body, where they have specific and different functions, depending on specific stimuli within their microenvironment (66). Based on *in vitro* experiments, macrophages can be broadly divided into two different functional and metabolic states: M1 and M2 macrophages (67). While M1 or pro-inflammatory macrophages are involved in inflammation and tissue destruction, M2 or anti-inflammatory macrophages are related with inflammation resolution and tissue repair (68). Although it is now clear that M1 and M2 macrophage classification is an oversimplification and represents the extremes of a heterogeneous cells with a very high level of plasticity, it is still useful today to evaluate the role of macrophages in different inflammatory context such as MS (69).

Under homeostatic conditions, macrophages in the CNS can be found in the meninges, perivascular space, and choroid plexus, which are referred to as CNS-associated macrophages (CAMs) (also known as border-associated macrophages (BAMs)) (70). CAMs are highly heterogeneous and can be further divided depending on their anatomical positions into meningeal macrophages (mμΦ), choroid plexus macrophages (cpμΦ), and perivascular macrophages (pvμΦ) (71). CAMs are constantly monitoring the CSF, searching for harmful antigens, and also contributing to the drainage of CNS-derived antigens (70, 72, 73). In pathological conditions, CAMs expand and secrete pro-inflammatory cytokines and chemokines that promote the recruitment of different immune cell population leading to neuroinflammation (52).

The first evidence that macrophages are important during the development of EAE came from a study showing that macrophage depletion with silica, a treatment that preferably depletes macrophages but not DCs, reduces severity and delays the onset of clinical symptoms when is administrated prior to EAE induction, and reduces EAE symptoms when is injected after the appearance of the first clinical signs (74, 75). By using mannosylated liposomes containing dichloromethylene diphosphonate to deplete macrophages, Huitinga et al. showed that intraperitoneal injection of the liposomes after the appearance of clinical symptoms efficiently depletes macrophages, drastically reducing EAE symptoms and diminishing the number of infiltrating macrophages into the CNS (76). Moreover, intraventricular injection of mannosylated clodronate liposomes, to specifically deplete CAMs, showed reduction of clinical symptoms in the EAE mouse model (77). However, these treatments failed to show specific macrophage depletion, since other APCs could also be affected (78, 79). Thus, new strategies were developed to overcome these difficulties. By using a combination of parabiosis experiment where two mice are joined together resulting in a shared circulatory system, and myeloablation, Ajami et al. found that monocyte infiltration and differentiation into macrophages are essential for the EAE progression and pathogenesis (80). Matrix metalloproteinase [MMP]-2 and MMP-9 are two proteins mainly produced by macrophages involved in leukocyte transmigration into the CNS (81). By using the MMP-2 and MMP-9 double knockout mice, Agrawal et al. showed that decreasing leukocyte transmigration and macrophage accumulation in the CNS reduces EAE symptoms, suggesting an active role of macrophages in neuroinflammation (81). Specific subsets of macrophage infiltration occur at different times during EAE progression. Thus, CNS is infiltrated with high levels of M1 macrophages during the onset of the disease, but there is a gradual increase in M2 macrophages during the recovery phase that is associated with improved neurological impairment (82). By using different strategies to either induce M2 macrophage polarization or directly injecting M2 macrophages, it has been showed that this anti-inflammatory macrophage population can reduce EAE symptoms (83–86). Due to the high heterogeneity of macrophage populations (CAMs and monocyte-derived macrophages) that infiltrate the CNS during EAE, new experimental technologies were developed to study the changes and contribution of these macrophage subsets during EAE progression. By using scRNA-seq analysis of the different immune cell types at different stages of EAE, Jordão et al. showed that during EAE, CAMs are transcriptionally distinct from their counterparts during homeostasis, demonstrating the plasticity of macrophages (52). Moreover, local proliferation of CAMs, with increased expression of MHCII, were evident during the onset of the disease, reaching the highest proliferation at the peak of EAE (52). However, MHCII ablation specifically in CAMs did not affect the development of EAE, suggesting that CAMs are redundant at least for antigen presentation in the CNS during EAE. All these results highlight the high heterogenicity of macrophage populations, with different involvement during EAE, but also suggest that treatments that favor M2 macrophage infiltration could be a good therapeutic target to treat neuroinflammation.

Macrophage infiltration into MS lesions was described over almost forty years ago (87). These macrophages express the inducible nitric oxide synthase (iNOS), a M1 marker, suggesting a proinflammatory phenotype (88). In an attempt to further characterize the phenotype of macrophages in MS lesions, Vogel et al, using a panel of typical M1 and M2 markers showed that myelin-laden macrophages in the demyelinated lesion area express high levels of the M1 markers CD40, CD86, CD64 and CD32 (89). Interestingly, M2 markers CD206 and CD163 were also strongly expressed by pvμΦ. Moreover, co-expression of CD40 and CD206 showed close to 70% of infiltrating macrophages positive for both markers, indicating an intermediate activation status (89). By using iron-sensitive magnetic resonance imaging, it was recently confirmed the increase in CD163 expression in myeloid cells from chronic brain active lesion of postmortem in pwMS (87). Applying imaging mass cytometry, Park et al. found the presence of macrophages phagocyting in active lesion of pwMS with differential phenotype depending on the position, from highly activated macrophages in the edges into less activated macrophages in the lesion center, that is in line with the simultaneous expression of pro- and anti-inflammatory markers by macrophages in MS lesions described by Vogel et al. (89). Thus, macrophages in the MS lesions are an heterogenous population with mixed pro- and anti-inflammatory phenotypes, capable of phagocyting myelin and interacting with other immune cells. Whether targeting macrophage population could alleviate MS symptoms needs to be addressed.

### Microglia

Microglia are highly specialized parenchymal-resident macrophages, important in mediating inflammatory and immune responses inside the CNS. While in developmental stages microglia regulates synaptic plasticity by modulating synaptic formation and elimination and shaping embryonic brain circuits, in adult stages microglia contributes to maintain homeostasis by participating in myelination and pruning processes or responding to pathological threats, acting as a first line of defense against pathogens or tissue injury, actively phagocyting and presenting antigens to T cells (90–92). Under homeostatic conditions, microglia are found in a resting state, characterized by a rod-shaped soma, several ramifications and decreased phagocytic conditions, while in an activated state microglia acquire an amoeboid shape, retracting their ramifications and increased their phagocytic and migratory capabilities (93). Microglia, in a similar way than macrophages, can be divided into a M1 and M2 population, although a continuum of intermediate phenotypes can be found (94). M1 microglia produces proinflammatory cytokines such as TNF-α, IL-6 or IL-1β and is involved in inflammation, while M2 microglia produces mainly IL-4 and IL-13 and is related to anti-inflammatory and healing processes (95). Because this dual role of microglia in inflammation and healing processes, these cells are considered to be a double-edged sword, where M1 microglia are necessary to fight against infection, but later M2 microglia need to expand to reduce inflammation and start healing processes, suggesting that a subtle balance in timing and expansion of M1 and M2 microglia are important for keeping the CNS homeostasis, and, as a corollary, a disbalance of both cell populations could contribute to neuroinflammatory and neurodegenerative disorders (96).

A relationship between microglia and MS came from early studies in animal models showing the presence of microglia in spinal cord lesions, together with the capacity of purified microglia to support an effector response by an encephalitogenic myelin basic protein-reactive CD4+ T cell line (97, 98). In fact, microglia activation occurs prior to the development of symptoms, suggesting that microglia are necessary for the development of EAE (99). First attempt to dissect the role of microglia in EAE came with a study by Heppner et al. where, by using a CD11b-HSVTK transgenic mice, all CD11b+ cells, including macrophages and microglia, express the herpes simplex thymidine kinase and after ganciclovir administration, it blocks cell activation, generating a “paralysis” of microglia and macrophages, that ameliorates EAE symptoms (100). However, because of the similarities between microglia and macrophages markers, it was difficult to specifically dissect the role of microglia during EAE progression. In line with this observation, the inhibition of the Colony-Stimulating Factor 1 Receptor (CSF1R) by the PLX5622, reduced both microglia and infiltrating-macrophage population and reduced EAE pathogenesis (101, 102). The use of scRNA-seq analysis as an alternative to overcome this limitation has shown heterogenicity of microglial populations both at homeostasis and during EAE (52). At the peak of the disease, microglia expand dramatically, although reducing MHCII expression in this population did not affect EAE progression, suggesting a redundant function of microglia on T cell activation (52). Recently, it has been shown that CD83 expression in microglia is important for regulating their function, by a study showing that specific deletion of CD83 expression in microglia promoted an over-activated phenotype, increasing the production of TNF and exacerbating EAE symptoms and neuroinflammation, suggesting that microglia, by modulating the microenvironment, could participate in the propagation of the neuro-inflammatory damage in the CNS during EAE (103). However, more studies are necessary to specifically dissect the role of microglia during EAE. Additionally, to the possible role in promoting inflammation and damage during EAE, some studies have suggested that microglia could be playing protective roles during the development of MS, by the production of immunosuppressive factors that could mediate myelin regeneration or myelin clearance, that ultimately leads to better recovery (104–106). An explanation for this dichotomous behavior could be attributed in part to different activation states; the rapid expansion of different microglia populations during disease; or the interaction with other immune cell types during neuroinflammation. The search of new specific markers to differentiate microglia from infiltrating macrophages, together with the development of new genetic or pharmacological approaches to specifically target microglia will be necessary to have a clear picture of the specific role of microglia during EAE.

Clusters of microglia, called microglia nodules, in brain lesions of pwMS have been described for over 30 years (107, 108). The appearance of these microglia nodules occurs before the MS lesion formation and persist throughout the entire course of the disease (109–112). Microglia nodules are highly phagocytic, produce inflammatory cytokines and radical oxygen species (ROS), which could contribute with axonal damage and degeneration (113–115). A recent study has tried to characterize the microglia nodules in MS lesions by using different genetic, molecular, and cellular approaches (116). Strikingly, microglia nodules are in areas with active axon demyelination; they have increased pro-inflammatory and ROS-related gene expression; they are in close contact with other immune cell types such as infiltrating macrophages; and correlated with severe MS pathology, suggesting that microglia nodules are participating in the initiation of lesions and the promotion of neuroinflammation in pwMS (116). Further studies considering the time course of the disease are necessary to confirm these findings, to consider microglia as a therapeutic target for MS.

### Neutrophils

Neutrophils are the most abundant leukocyte in the blood, representing around 40% to 70% of all white blood cells in humans and 10-25% in mice (117). Neutrophils are generated in the bone marrow and released into the blood, where they can live for some hours, around 10 h in mice and 18 h in humans, and they can be rapidly recruited to sites of infection, by following chemoattractant gradients to reach compromised tissues, working as a first line of defense against invading microorganisms (118–120). ROS production, phagocytosis, and the formation of neutrophil extracellular traps (NETs) are among the main mechanisms used by neutrophils to fight pathogens (121, 122).

Although it was thought that the antimicrobial activity was the main function of neutrophils, emerging evidence from the last 15 years has showed that neutrophils are able to produce cytokines, to interact with other immune cell types and also to express MHCII, suggesting that they can activate CD4+ T cells, modulating adaptive immunity (123–126). In fact, neutrophils can be found in non-inflamed tissues and in lymph nodes during homeostasis, suggesting that neutrophils can be also contributing to tissue homeostasis (125, 127). Due to its varied functions, several lines of evidence suggest that neutrophils dysfunction could be implicated in the pathogenesis of different autoimmune disorders including MS (128).

First evidence suggesting a role of neutrophils in EAE came from studies showing neutrophils accumulation in blood, spleen, peripheral lymph nodes, meninges and CNS during different stages of EAE progression (129–133). Blockade of neutrophil accumulation in circulation or neutrophil depletion, particularly at early stages, reduces EAE severity, suggesting a direct involvement of neutrophils in EAE progression (132, 133). Glutamic acid-leucine-arginine-positive (ELR+) chemokines (CXCL1, CXCL2 and CXCL6), which are produced by Th17 cells, and granulocyte-colony stimulating factor (G-CSF), produced by fibroblasts and epithelial cells after stimulation by Th17 cells, are important chemokines that mediate neutrophil recruitment into the CNS (133, 134). In the CNS, neutrophils seem to be involved in the blood-brain barrier (BBB) breakdown, and to support the activation of microglia and CNS-infiltrating macrophages, amplifying neuroinflammation (135, 136). NETs formation in the brain and spinal cords of animals suffering EAE has been also recently reported, which could facilitate the recruitment of Th1 and Th17 cells into the CNS (137). In the periphery, neutrophils seem to be important in the clonal expansion of autoreactive T cells in peripheral lymph nodes, as suggested by a recent study showing that reducing neutrophil accumulation in lymph nodes attenuate CD4+ T cell expansion and decreases EAE clinical score, by a mechanism dependent on TLR9 (132). Thus, neutrophils seem to contribute to EAE pathogenesis in different anatomical location; in the periphery, by activating and expanding autoreactive CD4+ T cells in peripheral lymph nodes and possibly the meninges, and in the CNS, by directly affecting BBB permeability, together with supporting the activation of microglia and CNS-infiltrating macrophages and promoting the recruitment of autoreactive T cells into the brain and spinal cord, all of which leads to neuroinflammation.

Initial studies about the role of neutrophils in pwMS were conflicting, with some studies showing increased neutrophil priming and neutrophil-producing molecules while other papers showed unaltered or even reduced neutrophil activity in peripheral blood from pwMS (138, 139). The difficulty of specifically analyzing neutrophils from other leukocytes in the blood could explain these contradictory results. In fact, the development of better neutrophil markers has confirmed elevated neutrophil count during MS clinical relapses compared to remission (140). Moreover, the neutrophil-to-lymphocyte ratio (NLR), has been found to be higher in blood from pwMS compared to healthy controls, although no differences between RRMS and progressive MS patients were found (141). A recent retrospective study confirmed the potential use of NLR as predictor of increased relapse rate and severity in MS (142). Blood neutrophils from RRMS patients have higher inflammatory markers, produce more pro-inflammatory cytokines and ROS and are resistant to apoptosis (143). Detection of neutrophils in the CSF of pwMS, particularly at early stages, suggests a direct involvement of neutrophils in the autoimmune damage (144). Additionally, increased levels of NETs and neutrophil elastase have been found in blood from pwMS compared to healthy volunteers, specifically in RRMS patients, suggesting an active role of neutrophils during inflammation (145). All these results suggest that neutrophils have a more activated phenotype in pwMS, and by different modes of action, involving increased production of pro-inflammatory cytokines, NETs formation, and ROS production, could contribute to the autoimmune damage in pwMS.

### Innate lymphoid cells

Innate lymphoid cells (ILCs) represent a subset of immune cells that share characteristics with classical lymphoid T cell subsets but are devoid of the antigenic presentation requirement for their activation. These cells express transcriptional regulators and effector cytokines similar to helper T cell subpopulations and, based on their molecular expression profile, are classified into three groups. ILC1s, which consists in conventional natural killer (NK) cells and so-called helper ILC1; both expressing the transcription factor T-bet and promoting type 1 immunity, are critical for controlling intracellular microbial infections and restraining tumor development. Functionally, NK cells have strong cytotoxic potential, expressing granzymes and perforins, whereas helper ILC1s produce much higher concentrations of IFN-γ than NK cells (146). Group 2 ILCs are dependent on the transcription factors GATA3 and RORα and produce the type 2 cytokines IL-4, IL-5, IL-9, and IL-13 (147–149). ILC2s plays protective roles in the expulsion of intestinal parasites and tissue repair, but also mediate detrimental host immune responses depending on timing, location, and physiological context. Interestingly, ILC2s, despite its scarcity, is the dominant innate lymphoid cell population in the lung, indicating a key role as first responder and amplifier upon immune challenge at this site (150). Group 3 ILCs includes fetal lymphoid tissue-inducer (LTi) as well as adult ILC3s, both depend on RORγt, but their distribution and functions are distinct. Specifically, LTi cells mediate the development of lymphoid tissues during embryogenesis via the production of lymphotoxin, while ILC3s is highly enriched in the gut, where they sense and integrate a wide range of cell-derived signals and environmental cues coming from microbiota and diet, shaping their phenotype and functions (151). ILC3s secrete different effector cytokines including IL-22, IL-17A, IL-17F, GM-CSF and lymphotoxin-α_3_ (LTα_3_) (147, 149, 152, 153). Intestinal ILC3s participates in the proper expression of tight junctions on gut epithelial cells, preventing the activation of innate immune cells through the stimulation of pathogen-associated molecular pattern receptors and avoiding the activation of autoreactive T cells by molecular mimicry (154–156). ILC-T cell interactions can contribute to immune tolerance by depleting commensal bacteria-specific T cells during homeostasis (157, 158), but also by stimulating antigen-specific T cells and pathogenic Th1 cell expansion in inflammatory conditions (158–161). Finally, ILCs are not only present on mucosal surfaces but indeed can be found in CNS during steady-state and inflammation. Even when ILC subsets were initially described as tissue-resident cells that proliferate locally, recent studies demonstrated that, in response to inflammation, gut ILCs acquire migratory patrolling attributes (162). ILCs can be recruited into extraintestinal tissues under inflammatory conditions, such as mesenteric lymph nodes, lung and CNS to promote an inflammatory T cell activation (163–165); however, the exact mechanism driving the recruitment of ILCs into the brain are still not well understood. Additionally, the role of ILCs in MS is still not completely understood as controversial findings have been reported assigning them either a protective or disease-accelerating role, depending on the analyzed subset.

Studies in mice models have shown that in homeostatic conditions, the three groups of ILCs reside both in the meninges and choroid plexus (166), giving CNS-resident ILCs an advantageous anatomical site to act as cerebral immune gatekeepers. Like their adaptive counterparts, Th2 cells have been described as having a neuroprotective role for CNS-resident ILC2s. Meningeal ILC2s has emerged as a novel regulator of microglial activation and BBB stability mediated by their IL-10 production (167). As MS pathology is well known to be sexually dimorphic, hormone differences between genders are associated to decreased levels of IL-33 and restricted ILC2 activation in a female transgenic mouse model of EAE, thus promoting increased susceptibility to developing the disease (168). Activation of ILC2s through IL-33 stimulation limits the Th17-dominated response characteristic of susceptible females and drives a non-pathogenic Th2 anti-myelin response. This evidence indicates that increased ILC2 function is associated with improved neurological outcomes in EAE. On the other hand, during EAE, both ILC1s and ILC3s are able to infiltrate the brain parenchyma, accumulating into the CNS. In particular, NK cells are directly involved in the process of demyelination (3). Nonetheless, the major fraction of CNS-infiltrating ILCs during disease belongs to RORγt^+^ ILC3s and this accumulation is largely due to cell recruitment rather than local proliferation, indicating migration from peripheral tissues (169, 170). ILC3s in the CNS uniquely expresses the trafficking receptors CCR6, CCR5, α4β7 and CXCR3, that are critical for the entry of lymphocytes into the inflamed CNS through the circulation (163). These receptors respond to ligands CCL20, CCL3/4, V-CAM1 and CXCL11, respectively, which are released from the CNS upon neuroinflammation (171). Interestingly, T bet-dependent NKp46^+^ ILCs (a group that includes NK, ILC1 and ILC3) controls the CNS parenchymal infiltration of myelin-reactive Th17 cells by generating a proinflammatory-cytokine environment in the meninges that is necessary for the reactivation and maintenance of IL-17A-producing CD4+ T cells in the CNS (172), consequently contributing to the propagation of neuroimmune response to CNS injuries. In addition to pro-inflammatory cytokines IL-17 and GM-CSF, meningeal ILC3s constitutively express CD30L and OX40L, denoting that ILC3s sustain neuroinflammation by supporting T cell survival and reactivation in the meninges (169). Inflammatory ILC3s derived from circulation infiltrates the CNS, are located in the proximity of T cells, and works as APCs that restimulate autoreactive T cells, performing complementary but non-redundant roles with conventional DCs that also act restimulating T cells after entry into the CNS (163). This complementary role might be regulated by the distinct localization of these cell types; ILC3s are enriched within focal lesions of the CNS parenchyma, while DCs are mostly concentrated at border-associated brain dura meninges and spinal cord leptomeninges. Collectively, this evidence indicates that ILCs are essential in CNS inflammation and reveals the potential of harnessing peripheral tissue ILCs for the prevention of MS.

Human studies have showed that NK cells participate in the process of demyelination, supporting the notion of a disease-accelerating role in this cell type. Saikali et al, showed that oligodendrocytes from pwMS express ligands for the activating NK cell receptor NKG2D that were not detected in healthy control samples, and that blocking NKG2D on NK cells significantly inhibited the killing of oligodendrocytes, suggesting a NKG2D-mediated killing mechanism for tissue injury in MS (173). Accordingly, EAE symptoms are reduced in NKG2D-deficient mice (174). In fact, NKG2D ligands, particularly UL16-binding protein 4 (ULBP4), was found to be highly expressed in active and chronic active lesions and normal-appearing white matter of pwMS compared to healthy controls (175). NKG2D ligands can also be shedding and soluble NKG2D ligands are elevated in the serum of pwMS (176). NK cells, CD4+ and CD8+ T cells seem to be the main immune cells involved in the killing mechanism mediated by NKG2D, although more experiments are needed to specifically dissect the contribution of each of these cell types in this process (177).

Epstein-Barr virus (EBV), a herpesvirus present in 90% of adults worldwide, has been considered to be an important factor in MS pathogenesis, mainly because molecular mimicry of EBV antigens and proteins expressed in glial cells that generate autoimmune responses (178, 179). However, only a small percentage of EBV-infected people develop MS, indicating that additional mechanisms should be compromised to develop MS in this population. Recently, it was shown that NK cells, particularly NKG2C+ and NKG2D+ NK cells, are capable of controlling autoimmune damage by killing autoreactive glial cells, and this process is dampened in pwMS (180). This study highlights a protective role of a subset of NK cells in MS pathogenesis, although more studies are necessary to clarify the specific role of different NK cell subsets in MS.

Cytomegalovirus (CMV), a herpesvirus, has a controversial role regarding MS pathogenesis, with a study associating CMV with lower MS susceptibility (181), while other study suggesting CMV infection with enhanced MS symptoms (182). NK cells, together with CD8+ T cells, are the main cells capable of eliminating CMV-infected cells (183). Accordingly, pwMS have increased NKG2C expression in NK cells compared to healthy donors, with the highest expression in the CMV-seropositive pwMS compared to CMV-seronegative pwMS, suggesting that CMV could promote NKG2C expression in NK cells, although the mechanism involved or the impact over NK cell function and MS pathogenesis are currently unknown (184). Thus, the relationship between CMV, NK cells and MS requires further studies.

Recent investigations showed a higher frequency of CD56^bright^ NK cells during relapse, indicating an immediate response to disease reactivation, while CCR6-related shifts among this population suggest altered ILC migration to the CNS during MS (185). CD56^bright^ NK cells are enriched in MS lesions and the choroidal tissue from MS donors and display an activated and migratory phenotype, similar to that of CD56^bright^ NK cells in the circulation (186). As such, the enrichment of CD56^bright^ NK cells in the CNS of pwMS could result from selective infiltration from the blood towards the brain. NK are not the only ILC subset with the ability to migrate into the CNS during MS, since ILC3s have also been detected in the cerebrospinal fluid of pwMS and the frequency of these cells positively correlates with the presence of higher lesions (163). Interestingly, both ILC1 and ILC3 subsets expressed Sphingosine-1-phosphate receptor 1 (S1PR1), which explains their reduced absolute numbers in the peripheral blood of RRMS patients treated with Fingolimod, a sphingosine 1-phosphate receptor modulator used as a first-line therapy for RRMS, compared with treatment-free pwMS (187), suggesting that S1PR1 agonist sequesters peripheral blood human ILCs in lymph nodes. Collectively, these data suggest that ILC1 and ILC3 subsets migrate to the CNS and promote cytotoxicity and inflammation in pwMS.

### Mast cells

Mast cells (MCs), first described by Paul Ehrlich in 1878, are multifunctional and heterogeneous cells characterized by the expression of CD117, ST2 (also known as Interleukin 1 receptor-like 1) and the high affinity receptor for immunoglobulin (Ig) E (FcϵRI), and classically involved in a IgE-dependent allergic responses against parasitic invasion. They circulate as progenitor cells and populate different tissues, where they become mature MCs by interacting with local stem cell factors. Thus, MCs are located at sites of contact with antigens and allergens: skin, around blood vessels, bronchioles, mucous glands, and gut (188, 189). Upon activation, MCs release granules containing histamine, proteases (tryptase and chymase), prostaglandins, heparin, leukotrienes, cytokines, chemokines, and growth factors (190, 191). MCs are also located in the CNS, particularly in the leptomeninges, dura mater, choroid plexus, parenchyma of the thalamic-hypothalamic region, and cerebral side of BBB, suggesting that they could be involved in CNS pathologies (192, 193). Under physiological conditions, MCs maintain bidirectional communication with neurons so that MCs-heparin secretion blocks calcium flow, reducing neuronal communication, while neurons, through the secretion of neuropeptides, stimulate adhesion, degranulation, and secretion of cytokines and chemokines by MCs (191).

The role of MCs in the pathogenesis of EAE has been challenging due to their non-circulating state in the blood, low representation in tissues, and manipulation. Initially, a deleterious role of MCs in EAE arose from studies using the mast-cell-deficient Kit^W/W-v^ mouse model, that showed reduced clinical symptoms and delayed onset of EAE, effect that was reverted by the transplantation of bone-marrow derived MCs (194). Interestingly, bone marrow derived MCs transplantation failed to repopulate MCs in the CNS, suggesting that peripheral MCs are important mediators in EAE (195). Even though this early evidence, the use of a different MC-deficient mouse model, the Kit^w-sh^ mice, showed exacerbated EAE disease and increased T cell response against myelin (196). The discrepancies between these two models of MCs deficiency, could be explained in part by the different immunization protocols, since the same group showed that Kit^W/W-v^ animals showed reduced EAE symptoms at high, but not low doses of antigen and adjuvants (196). Further studies showed a pathogenic role of MCs located in the meninges, by contributing to the infiltration of T cells into the CNS, in a process dependent on the production of TNF by MCs (197). High numbers of MCs are found in the meninges, and Kit^W/W-v^ mice showed reduced T cell and neutrophil infiltration into the meninges and brain parenchyma, a process that can be reverted by intracranial mast cell reconstitution (197). Other studies have shown that MCs could be involved in the activation of different immune cells through the production of proinflammatory cytokines and chemokine secretion such as MCP-1, IL-6, and IL-33 (190, 198). MCs could also act as APCs, establishing a fine bidirectional communication with T cells, where MCs degrade myelin through their proteases and present antigens to T cells, which in turn stimulate MCs to secrete MMP-9, IL-6, and proteases, enhancing myelin destruction (199). Moreover, it has been demonstrated that MCs, upon activation of the NLRP3 inflammasome, secrete IL-1β, which induces the release of GM-CSF by T cells, a cytokine that enhances EAE pathogenesis (200). Failure in NLRP3 function reduces demyelination and promotes resistance to the development of EAE (193). Additionally, MCs, by secreting IL-4, IL-10, IL-13, TGF-β, TNF-α, and IL-6, can promote T cell differentiation into Th1, Th2, and Th17, while in an OX40L-dependent way, MCs can suppress Treg function (190, 193, 201). It has also been demonstrated that MCs interact with Th9 cells, which have been implicated in the pathogenesis of EAE, and that through the secretion of IL-9, they recruit MCs from the spleen to the CNS, indicating that not only resident MCs but also peripheral MCs are associated with EAE (202).

It seems that MCs have a dual role inside CNS in humans, since the secretion of IL-6 by MCs at low concentrations is neuroprotective and stimulates the proliferation of neuronal stem cells; however, at high concentrations, it promotes neurotoxicity (203). Similarly, the binding of TNF-α to its Tumor necrosis factor receptor (TNF-R) 2 receptor induces neurogenesis, while TNF-R1 activation is associated with neurodegeneration (191). In addition to producing cytokines, MCs produce approximately 20-40% of cerebral serotonin, a neurotransmitter that may play a dual role in MS depending on which receptor it binds to, since H2Rs and H3Rs are neuroprotective, while H1Rs and H4Rs are inflammatories (204). MCs also promote angiogenesis through the secretion of VEGF, FGF-2, and Osteopontin (OPN), which in turn stimulates VEGF secretion establishing synergy. Thus, MCs promote increased recruitment of immune cells to the CNS (205). Therefore, depending on the immune context and inflammatory environment, MCs can act as detrimental or protective agents in MS. In summary, the use of new MCs-deficient mice models is necessary to help clarify the exact role of MCs during MS.

### Basophils

**Figure 1:**
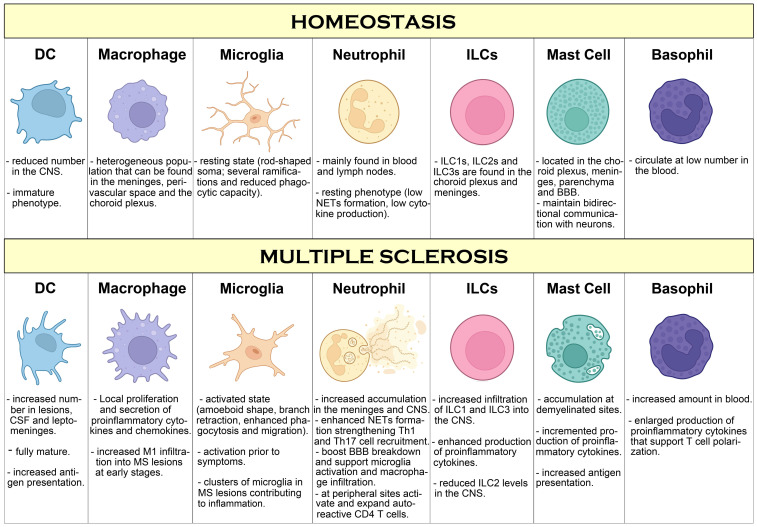
Overview of innate immune cells and pathways compromised during MS. During homeostasis conditions, besides microglia covering the brain and spinal cord in a resting state, few innate immune cell types can be found in the CNS, mostly in the meninges and choroid plexus where they work sensing and controlling possible threats such as pathogen infection. However, during MS, increased infiltration of innate immune cells in perivascular regions and in the brain parenchyma is observed, promoting inflammation, immune cell infiltration and T cell activation that leads to neuroinflammation, neurodegeneration and myelin damage. Additionally, innate immune cells in the periphery support inflammation by activation and polarization of autoreactive T cells, that further contribute to MS pathogenesis.

Basophils are short-lived innate immune cells characterized by the presence of basophilic granules in the cytoplasm, representing less than 1% of all leukocytes in the peripheral blood. Basophils share several features with mast cells, including the expression of the FcϵRI, but basophils express high level expression of IL-3 R alpha/CD123, and they do not express CD117/c-kit (206). Basophils, by expressing MHCII and co-stimulatory molecules, are capable of presenting antigens to CD4+ T cells and are believed to be important in promoting Th2 cell differentiation through the production of IL-4 (207). However, additionally to IL-4, basophils can produce IL-6, a cytokine important in mediating a Th17 response, suggesting that they are important mediators in Th17 cell-mediated immune responses (208). The role of basophils in EAE has been first drawn by a seminal study showing that basophils, by actively secreting IL-6, support Th17 polarization by DCs (209). Because Th17 cells are important mediators in EAE, this study suggests that basophils could contribute to the autoimmune damage by indirectly supporting Th17 cell polarization. In fact, by using basophil-deficient mice, the authors found reduced EAE clinical score together with a reduction in the number of CD4+ T cells that infiltrate CNS and decreased levels of IL-17 production by these cells (209). This study suggests a direct role of basophils in promoting autoimmune damage in EAE, although more research is necessary to confirm and expand these findings. Moreover, elevated number of basophils have been observed in the blood of pwMS compared to healthy controls, although the significance of this observation over the development or progression of MS remains unknown (210). Thus, more studies are necessary to understand the role of basophils during MS. A summary of innate immune components involved in MS pathogenesis is shown in **Figure 1**.

## Conclusions and perspectives

MS is a complex disease not only because there is no cure for it, but also because its cause is unknown. MS is therefore a disease that presents several challenges in its diagnosis, treatment and management. Some of the key challenges and future directions lie not only in effective and personalized treatment, but in early and accurate diagnosis. While there are treatments to manage the symptoms of MS, there is no known cure. Hence, it is necessary to have a better understanding of the inflammatory processes that contribute to the development of this disease, as well as the modulation of these processes. In this sense, a complex interplay between immune cells coordinates the cascade of inflammatory events that contributes to development of the disease in MS. Adaptive immunity has been studied extensively over the years, but less emphasis has been placed on innate immune changes that occur in MS. As a result of this, current approved MS therapies primarily target peripheral lymphocytes and thus are mainly effective in treating RRMS. In progressive MS, where the adaptive immune response plays a less prominent role, microglia and CNS-associated macrophages are activated in a pro-inflammatory phenotype that promotes demyelination and neurodegeneration. However, to date, there are no specific therapies targeting primarily innate immune cells in MS. As the role of innate immune cells in MS becomes better described (see **Table 1**), it will be possible to design novel approaches to therapeutically target both central and peripheral innate immunity to promote remyelination, reduce neuroinflammation and increase CNS repair in pwMS.

**Table 1:** Pathogenic and protective roles of innate immune cells in multiple sclerosis (MS) and experimental autoimmune encephalomyelitis (EAE).

Cell type	MS	EAE
Dendritic cells	Accumulation of different DCs subsets in perivascular regions of the CNS contributing to the increase of neuroinflammation by actively presenting myelin antigens to T cells (60–64).	cDC2 are necessary for T cell activation to promote EAE pathogenesis (51). Levels of MHCII on cDCs correlates with disease severity (52).
Macrophages	In MS lesions exists an heterogenous populations of macrophages with pro- and anti-inflammatory phenotypes (89).	MMP produced by macrophages promotes leukocyte transmigration into the CNS (81). M1 infiltration is higher in disease onset, while M2 increases during recovery phase of the disease (82).
Microglia	Microglia nodules are present in areas with active axon demyelination, promoting neuroinflammation in MS patients (116).	By modulating the microenvironment, microglia participate in the propagation of the neuroinflammatory damage in the CNS (103).
Neutrophils	In MS patients present a more activated phenotype that contributes to autoimmune damage (143, 145).	Involved in the BBB breakdown, activation of microglia and CNS-infiltrating macrophages (135, 136) and the recruitment of Th1 and Th17 cells to CNS (137).
Innate Lymphoid Cells	NK cells have dual role, a disease-accelerating role in MS by participating in the process of demyelination (173), and a protective role by killing autoreactive glial cells (180).	Increased ILC2 function is associated with improved neurological outcomes in EAE (167, 168). ILC1s and ILC3s promote autoimmune response by controlling CNS parenchymal infiltration of Th1 and Th17 cells (172).
Mast cells	Depending on the immune context, inflammatory environment and magnitude of the response, MCs can act as detrimental or protective agents in MS (203–205).	Mast cells promote EAE pathology by contributing to the infiltration of T cells into the CNS in a TNF-dependent manner (197), enhancing myelin destruction (199) and suppressing Treg function (190, 193, 201).
Basophils	Elevated number of blood basophils in pwMS compared to healthy controls, with unknown relevance for MS progression (210).	Basophils contribute to the autoimmune damage by indirectly supporting Th17 cell polarization (209).

Finally, a multidisciplinary approach incorporating collaboration between neurologists, immunologists, geneticists and other experts could certainly help advance the understanding of MS and develop more effective treatments that involve not only neuroinflammation but also neurodegeneration and repair.
